# Factors associated with the retention of *travestis* and transgender women living with HIV in a peer navigation intervention in São Paulo, Brazil

**DOI:** 10.1590/0102-311XPT147522

**Published:** 2023-05-01

**Authors:** Katia Cristina Bassichetto, Gustavo Santa Roza Saggese, Luca Fasciolo Maschião, Paula Galdino Cardin de Carvalho, Hailey Gilmore, Jae Sevelius, Sheri A. Lippman, Maria Amelia de Sousa Mascena Veras

**Affiliations:** 1 Faculdade de Ciências Médicas da Santa Casa de São Paulo, São Paulo, Brasil.; 2 University of California, San Francisco, U.S.A.

**Keywords:** Transgender Persons, Transvestism, HIV Infections, Vulnerable Populations, Longitudinal Studies, Mulher Transgênero, Travestis, Infecções por HIV, Populações Vulneráveis, Estudos Longitudinais, Mujer Transexual, Travestido, Infecciones por VIH, Poblaciones Vulnerables, Estudios Longitudinales

## Abstract

*Travestis* and transgender women (TrTGW) constitute the groups with the highest HIV prevalence in the world, with higher probability of infection compared with the general population and lower adherence to prevention and treatment strategies than other vulnerable groups. Considering these challenges, this study describes the factors associated with the retention of TrTGW with HIV to the TransAmigas project. Participants were recruited from April 2018 to September 2019 in a public health service in São Paulo, Brazil. A total of 113 TrTGW were randomly assigned to either a peer navigation intervention (75) or a control group (38) and followed up for nine months. To analyze the association between the selected variables and the outcome (“retention at nine months”, regardless of contact at three months, defined by the “full completion of the final questionnaire”), bivariate and multivariate logistic regression models were used. Peer contact forms were qualitatively assessed to validate and complement the previous selection of quantitative component variables. Of the 113 participants, 79 (69.9%) participated in the interview after nine months, of which 54 (72%) were from the intervention group and 25 (66%) from the control group. In the final multivariate model, contact at three months (adjusted odds ratio – aOR = 6.15; 95% confidence interval – 95%CI: 2.16–17.51) and higher schooling level (≥ 12 years) (aOR = 3.26; 95%CI: 1.02–10.42) remained associated with the outcome, adjusted by race/skin color, age ≤ 35 years, and HIV serostatus disclosure. Future studies with TrTGW should include contact at regular intervals, with additional efforts aimed at participants with lower schooling level.

## Resumo

Travestis e mulheres trans (TrMT) pertencem aos grupos com mais alta prevalência do HIV no mundo, com maior probabilidade de infecção em relação à população geral e menor adesão a estratégias de prevenção e tratamento do que outros grupos vulneráveis. Considerando esses desafios, descrevemos os fatores associados à retenção de TrMT com HIV no projeto TransAmigas. O recrutamento ocorreu entre abril de 2018 e setembro de 2019, em um serviço público de saúde em São Paulo, Brasil. Foram inscritas 113 TrMT, atribuídas aleatoriamente para uma intervenção com navegadora de pares (75) ou um grupo controle (38) e seguidas durante nove meses. Para analisar a associação entre as variáveis selecionadas e o desfecho (“retenção aos nove meses” independentemente do contato aos três meses, definido pela “resposta completa ao questionário final”), foram empregados modelos de regressão logística bi e multivariados. Uma exploração qualitativa dos formulários de contato de pares foi realizada para validar e complementar a seleção prévia de variáveis do componente quantitativo. Das 113 participantes, 79 (69,9%) responderam à entrevista de nove meses, sendo 54 (72%) do grupo intervenção e 25 (66%) do grupo controle. No modelo multivariado final, o contato aos três meses (odds ratio ajustado – ORa = 6,15; intervalo de 95% de confiança – 95%CI: 2,16–17,51) e a maior escolaridade (≥ 12 anos) (ORa = 3,26; IC95%: 1,02–10,42) permaneceram associados ao desfecho, ajustados por raça/cor, idade < 35 anos e revelação do status sorológico para HIV. Pesquisas futuras com TrMT devem incluir contato em intervalos regulares, com esforços adicionais voltados a participantes com menor escolaridade.

## Resumen

La población de travestis y mujeres trans (TrMT) está en los grupos con mayor prevalencia de VIH en el mundo, con mayor probabilidad de infección en comparación con la población general y menor adherencia a las estrategias de prevención y tratamiento que otros grupos vulnerables. Ante estos desafíos, describimos los factores asociados a la retención de TrMT con VIH en el proyecto TransAmigas. La selección ocurrió entre abril de 2018 y septiembre de 2019, en un servicio público de salud de São Paulo, Brasil. Se inscribieron 113 TrMT, a las cuales se asignaron aleatoriamente a una intervención de navegador de pares (75) o a un grupo control (38) y se les dio seguimiento durante 9 meses. Para analizar la asociación entre las variables seleccionadas y el resultado (“retención a los nueve meses” independientemente del contacto a los 3 meses, definido por “respuesta completa al cuestionario final”), se utilizaron modelos de regresión logística bi- y multivariante. Se realizó una exploración cualitativa de los Formularios de Contacto de Pares para validar y complementar la selección previa de las variables en el componente cuantitativo. De las 113 participantes, 79 (69,9%) respondieron a la entrevista de los 9 meses, de las cuales 54 (72%) pertenecían al Grupo Intervención y 25 (66%) al Grupo Control. En el modelo multivariante final, el contacto a los 3 meses (odds ratio ajustado – ORa = 6,15; intervalo de 95% de confianza – IC95%: 2,16–17,51) y un alto nivel de instrucción (≥ 12 años) (ORa = 3,26; IC95%: 1,02–10,42) permanecieron asociados con el resultado, ajustado por etnia/color, edad < 35 años y divulgación del estado serológico respecto al VIH. Los futuros estudios con la población TrMT deberán incluir contacto a intervalos regulares, con esfuerzos adicionales dirigidos a las participantes con menor nivel de instrucción.

## Introduction

*Travestis* and transgender women (TrTGW) are among the key populations with the highest HIV prevalence, accounting for 70% of HIV infections worldwide in 2021^1^. TrTGW not only have a disproportionate probability of infection compared with the general population, with a 14-fold increased risk compared with cisgender women^1,2^, but also a lower adherence to prevention and treatment strategies than other vulnerable groups^3,4^. Their experiences, permeated by stigma and discrimination, are often crossed by social markers, such as class and race, that make belonging to a minority group even more challenging and promote generally less favorable health outcomes^5^.

Besides the stigma faced daily, TrTGW have specificities that have not been sufficiently met, such as care related to gender issues, the need to be called by social name, and care at alternative periods in health services^6,7^. Another problem concerns the other health issues experienced, leading this group not to consider HIV diagnosis as a priority^8^. Therefore, HIV infection in TrTGW should be seen as a syndemic^9^.

In Brazil, the AIDS detection rate fell from 21.4 cases per 100,000 population in 2012 to 18.0 in 2019^10^. Since 1996, the Brazilian Unified National Health System (SUS) guarantees universal and free access to HIV treatment, including appointments with infectious disease specialists and other specialized professionals^11,12^. This initiative made Brazil, at the time, a pioneer in the fight against the AIDS epidemic^13^. However, despite the reduction in the AIDS detection rate in the general population, some vulnerable groups, such as TrTGW, still have difficulty initiating treatment, and, when they do, they often fail to maintain adherence^14^, partly due to a combination of factors, such as the stigma related to gender identity, extreme social inequality, and the insufficiency of inclusive public policies in education and health. This unfavorable context promotes high school dropout rates and affects access, linkage, and retention to health services^3,15^.

Some strategies to overcome these barriers have been proposed, with the potential to meet, even if only partially, the specific needs of TrTGW, especially regarding HIV prevention and treatment. As this group has difficulty accessing and low adherence and retention to health services, combined social-structural interventions that considered what makes this population less likely to adhere to treatment have been developed. Among these interventions, we highlight sensitization and training of health professionals for the development of specific skills, social support, and interventions focused on gender affirmation, such as the expansion of access to hormone therapy integrated with HIV treatment, aiming to promote autonomy and reduce stigma and discrimination in health services^3,16,17,18^.

Systematic reviews conducted in several continents with TrTGW living with HIV^2^ and, especially, a study with rural populations living with HIV in South Africa^19^ served as the basis for the development of the TransAmigas project. This project aimed to evaluate the effectiveness of peer navigation in increasing adherence to antiretroviral therapy (ART) among TrTGW. Peer navigation interventions are strategies in which individuals who are part of the same key population, with similar profiles, follow up the participants for a certain period to help them with their health-related issues. This strategy has proven important to improve HIV-related outcomes by providing an opportunity to focus on patients’ personal realities beyond health services, which contributes to overcome barriers and facilitates treatment adherence and retention^20^.

This project was conducted in São Paulo, the most populous city in Brazil, with more than 12 million inhabitants. This city has a high Human Development Index (HDI)^21^, but about 85% of its inhabitants live in areas of social exclusion^22^.

São Paulo is home to an important community of homosexual, bisexual, and transgender people. Since 1997, in partnership with public authorities and private institutions, the social movement annually holds one of the largest LGBT+ pride parades in the world, which surpasses one million participants since its seventh edition in 2003^23^. Same-sex marriage was legalized in the country in 2013^24^ and, since 2018, transgender people have the right to legally change their names^25^. However, as in the rest of Brazil, TrTGW living in São Paulo face great social marginalization and have difficulty adhering to health care^26^.

The living and health conditions of this group have promoted the development of studies in Brazil^27,28^, including in São Paulo^26,29^. Aiming to expand the knowledge about the characteristics and needs of TrTGW, TransAmigas project innovated by providing a personalized and culturally targeted intervention for this group, using as a reference the gender affirmative model, developed by Sevelius et al.^30^ (p. S65), which defines gender affirmation as “*an interpersonal, interactive process whereby a person receives social recognition and support for their gender identity and expression*”. The gender affirmative model was developed to understand why TrTGW are disproportionately affected in their living conditions and health and how the gender affirmation process relates to risk behaviors, self-care, and search for health services. Thus, this project considers that multidimensional phenomena, such as the deprivation of rights, racism, sexism, and transphobia, converge to widen social inequalities and bring potentially preventable damage to health. On the other hand, negative health outcomes decrease as gender affirmation needs are met by adequate support^30,31^.

Considering these challenges, this study presents the factors associated with the retention of TrTGW living with HIV to the TransAmigas project.

## Methods

The project *Improving Health Outcomes for HIV-positive Trans Women in Brazil* (TransAmigas project) was conducted as a pilot to test the feasibility and acceptability of a peer navigation intervention aimed at improving adherence to ART among TrTGW^32^. This cohort study was performed in the city of São Paulo in a SUS health service called STD/AIDS Reference and Training Center (CRT-DST/Aids SP). The expected sample size was 150 participants.

Participants were recruited from April 2018 to September 2019, using a convenience sample. In total, 194 potential participants were identified by different initiatives, of which the most effective was inviting CRT-DST/Aids SP users and participants of previous research coordinated by the Center for Research on Human Rights and Health of the LGBT+ Population (NUDHES). External actions were also performed, such as the distribution of printed invitations in spaces of sociability of TrTGW by the peer navigators (PNs) themselves (TrTGW who also lived with HIV). Finally, health professionals who worked with homeless people helped disseminate the research. Among the potential participants, 32.4% (45/194) were excluded for several reasons, such as: not agreeing to schedule an interview; not attending the eligibility confirmation; not being located after telephone contact; or not meeting all eligibility criteria at the time of the interview (being under 18 years old; not living in municipalities in Greater São Paulo; and/or not wanting to enroll in the CRT-DST/Aids SP for HIV treatment).

Of the remaining 149, 14 missed the face-to-face screening, 14 were not eligible, and eight refused to participate in the study. Finally, 113 TrTGW^32^ were randomly assigned to either the peer navigation intervention (75) or the control group (38), in a 2:1 ratio.

### Procedures

The protocol established that the participants in the intervention group would attend at least one monthly face-to-face meeting with their respective PN, holding individual thematic sessions on self-esteem, self-care, communication strategies, and ART adherence, over nine months. All participants had to be undergoing follow-up for HIV at their partner health service.

At enrollment, a baseline questionnaire was applied face-to-face by trained interviewers. After three months, participants received a phone call to confirm contact, check the quality of the intervention, and be reminded to return at nine months to complete the follow-up questionnaire, either face-to-face or remotely, ending their participation in the project. The initial protocol estimated 70% retention in this stage^32^.

To facilitate retention to the project, all participants received a BRL 30.00 compensation at the time of the interviews, regardless of the group to which they were randomized, to cover transportation and food expenses. Moreover, at enrollment, they received a small snack and a kit with beauty products, condoms, an enema, a lubricant gel, and a guide to services for TrTGW, besides BRL 20.00 per month to add minutes on their cell phones to maintain contact with the research team. As an additional incentive for the participants of the intervention group to attend the face-to-face meetings, PNs distributed small gifts, such as pill boxes, appointment books, stylized panties, among others.

### Analysis

A quantitative analysis was performed and its outcome was “retention at nine months”, regardless of telephone contact at three months. Retention to the project was defined by the “full completion of the final questionnaire” applied at nine months, which could be answered face-to-face or remotely. Therefore, it is an object cut-out of the original study. This definition is restricted to the contact between the participants and the research team, without considering retention to health care or peer navigation procedures. However, to meet the main objective of the project, clinical data were collected to identify HIV treatment adherence, defined by at least two medical visits or ART abandonment, with a maximum six-month interval during the research period^32^. These data are not part of the scope of this study.

To determine the variables associated with retention to the project, several variables of the baseline questionnaire included in the gender affirmative model were considered, such as sociodemographic and behavioral information and data on gender affirmation, mental health, substance use, and experiences of transphobia.

“Name change on personal documents” was the gender affirmation variable (“Yes, I did”; “I tried, but I couldn’t”; “I’m trying; I’ve already filed the lawsuit”; “I haven’t tried, but I intend to try”; “I haven’t tried”). A normalized four-item score was also created to synthesize a measure of internalized gender affirmation: (1) “Generally speaking, I feel good about myself and my gender identity”; (2) “I fully accept myself in my gender identity”; (3) “I’m proud of being a *travesti*/trans women”; and (4) “My feelings about my gender identity are mostly positive”. These items describe feelings regarding participants’ own gender identity, which may affect their decision or ability to remain engaged in both the project and health care. The answer options were based on the Likert scale, ranging from 1 (“strongly disagree”) to 5 (“strongly agree”).

The following variables of mental health and use of alcohol and other substances were selected: “psychic distress”, measured using the K10 scale^33^ (likely to be well; likely to have a mild disorder; likely to have a moderate disorder; likely to have a severe disorder); “frequency of alcohol use” (never; once a month; 2–4 times a week; 2–3 times a week; ≥ 4 times a week); “illicit substance use in the last six months” (no; yes); “prior suicide attempt” (no; yes); and “HIV serostatus disclosure to someone other than health professionals” (no; yes).

Regarding experiences of transphobia, the following variables were selected: “previous experience of verbal violence for being trans” (no; yes) and “previous experience of sexual violence for being trans” (no; yes). The participants’ “allocation group” (control group; peer navigation intervention group) and their response to the “check telephone call at three months” (no; yes) were also outcome predictor variables.

In order to evaluate the effect of the stigmatization process on the retention of TrTGW, peer contact forms were qualitatively analyzed, considering the questions previously used in the quantitative component as search criteria. The forms were completed by the PNs to record both the attempts to contact the participants and the topics discussed in the meetings, such as their location and the participant’s mental state and appearance. This information was typed and exported to a spreadsheet, allowing the identification of the following thematic categories: schooling level; financial situation; marital status/relationships; occupation/professional situation; general health; mental health; alcohol and other drug use; HIV-related issues; and family and housing situation. The category “additional information” was also included, with other themes that caught the attention of the PNs.

These categories were later divided into analytical subcategories, such as abusive relationships, financial difficulties, and lack of family support regarding gender identity. In turn, these subcategories were evaluated for the recurrence in which they appeared in the forms. Since the information reflected a follow-up history of up to nine months, the transformations experienced by the participants throughout the process could be observed.

### Statistical analysis

For quantitative variables, the absolute and relative frequency of the selected categories were estimated and the mean and standard deviation were used as measures of central tendency and dispersion for continuous variables, respectively.

To analyze the association between the selected variables and the outcome, bivariate and multivariate logistic regression models were used. Analyses were performed in Stata 13 (https://www.stata.com). The construction of the final model included variables based on the pre-existing literature, regardless of statistical adjustment criteria, variables with significant association in the bivariate analysis (p < 0.05), and the variable “name change on personal documents,” evidenced by qualitative analysis^34,35^.

For bivariate regression models, odds ratios (OR) were estimated for each category of predictor variables, while multivariate models considered adjusted odds ratios (aOR), 95% confidence intervals (95%CI), and p-value. The following categories of predictor variables were aggregated: schooling level (< 12 and ≥ 12 years of complete education); age (< 35 and ≥ 35 years old); and race/skin color (“other” referring to Asian and indigenous women). For statistical decision-making, in the final model, variables with p < 0.05 were considered significant.

### Ethical aspects

The TransAmigas project was approved by the Research Ethics Committee of the University of California, San Francisco (USSF), United States (16–19922), on October 4, 2016, and the Brazilian National Research Ethics Commission (CAAE: 61338116.0.0000.5375), on July 13, 2017. Potential participants were previously interviewed to confirm their eligibility and informed about the planned procedures before signing the informed consent form, thus, they had the right to withdraw from the study at any time, without any losses.

## Results

The mean age of participants was 32.9 years old (standard deviation – SD = 10.2); 44.3% were aged 25–34 years. Most participants (59.3%) were black or mixed-race. In total, 84.1% had disclosed their serostatus to someone other than health professionals; 63.9% reported moderate or severe mental health problems; 66.4% had used illicit substances in the last six months; 39.8% had already attempted suicide; 45.1% had experienced sexual violence; and 92% had experienced verbal violence for being TrTGW. Regarding gender affirmation, 88.5% agreed or strongly agreed that their appearance corresponded to their gender identity (Table 1).

Of the 113 participants, 83.2% (94/113) were located for the scheduling of the interview after nine months. Of these, 90.4% (85/94) agreed, but did not attend, even after multiple rescheduling attempts. Finally, 92.9% (79/85) actually participated in the interview, corresponding to 69.9% retention to the study. Of the 79 respondents, 72% (54/75) were from the intervention group and 66% (25/38) from the control group. About half of the interviews were face-to-face and the rest were via the Internet or by telephone.

In the bivariate logistic regression model, retention at nine months was associated with: “response to the check telephone call at three months” (OR = 7.78; 95%CI: 2.96–20.44); “higher schooling level” (≥ 12 years of complete education) (OR = 3.53; 95%CI: 1.31–9.47); and “illicit drug use in the last six months” (OR = 0.4; 95%CI: 0.16–1.03) (Table 2).

In the final multivariate model, “contact at three months” (aOR = 6.15; 95%CI: 2.16–17.51) and “higher schooling level” (≥ 12 years) (aOR = 3.26; 95%CI: 1.02–10.42) remained associated with the outcome, adjusted by race/skin color, age under 35 years, and HIV serostatus disclosure. The pseudo R2 value was 0.1880 (Table 3).

## Discussion

Considering the challenges of recruiting and retaining TrTGW living with HIV, which is a highly vulnerable population, the retention indicator in the TransAmigas project reached the expected value, according to what was hypothesized during its preparation. Several intervention studies have been using these indicators to assess factors that may interfere in the acceptability and feasibility analyses, especially in stigmatized populations^36^. Studies conducted in sub-Saharan Africa^37,38,39,40^, the United States^41^, and Brazil^42^ with men who have sex with men (MSM) and TrTGW presented great variability (50–93%) in these indicators, depending on the design, type, and characteristic of the population, location, and context. As a result, estimation methods vary from methods that consider intermediate visits to approaches with non-binary categorical outcomes.

In this study, we found a statistically significant association between retention at nine months and schooling level greater than or equal to 12 years and response to the check telephone call at three months. In studies with diverse populations, schooling is consistently associated with retention^41^, suggesting the existence of structural factors that do not exclusively affect TrTGW. In Brazil, basic education, offered free of charge by the government, comprises 12 years of complete education (primary and secondary). However, this factor should be interpreted in the light of other structural determinants. Especially in the case of TrTGW, low access and permanence are influenced by the effects of biographical background, family support, experiences of transphobia, and the need for early work^43,44^. Therefore, schooling is an independent predictor of retention for a better understanding of procedures, greater organization for return, and a social position that allows the expenditure of time and displacement that retention requires. At the same time, it is among the factors that explain greater retention in studies with TrTGW and MSM and characterizes the perception of individuals of greater safety and openness provided by the research environment to talk about their own sexuality^37,38,42^.

The association between the outcome and response to the check telephone call at three months shows that this strategy increases retention in longitudinal studies, considering the greater contact with the research team, regardless of the intervention. On the other hand, it may suggest a confluence of other factors that influence the response to the telephone call, such as housing stability, fixed income, participation in the labor market, access to a personal cell phone, and property security, even if these conditions have not been associated with the outcome. Some studies point having a cell phone and using social media, as well as the existence of safe spaces and researchers properly trained to establish bonds, important factors for greater retention of hard-to-reach populations^36,39,45^. A study on pre-exposure prophylaxis (PrEP) among MSM and TrTGW, with multiple visits, observed that early contact was a significant predictor of long-term returns^42^. This finding may be analogous to the effect of early telephone contact in the TransAmigas project.

We found no association between the outcome (“retention to the study at nine months”) and the variables study type, race/skin color, age, and name change on personal documents. The non-association between being part of the intervention group and greater retention suggests the need to treat retention to the project and retention to the intervention differently. Although the calculation of retention to the intervention and the evaluation of associated factors are outside of the scope of this study, the different contexts in which the intervention took place and the research procedures support this distinction. While the intervention was conducted by TrTGW living with HIV, the recruitment, enrollment, and interviews were performed in a health service by a team of cisgender interviewers with higher education. Moreover, only visits that occurred within the research period (baseline and at nine months) were reimbursed. On the other hand, peer navigation requires creating bonds and, therefore, more frequent contacts. They also allow the sharing of experiences potentially unique to this population.

The variables age and race/skin color, in turn, had no significant effect in the TransAmigas project, although they were classically associated with higher retention in longitudinal studies, possibly due to the sample size. They were also not significantly associated with the outcome “name changed on personal documents”, which is a right legally guaranteed in Brazil. Discrimination and disrespect to the changed/preferred name exists at all levels of health care^17^, which makes them barriers for this group to access services and a predictor of worse mental health^46^. The welcoming environment and the research team, which was sensitive and capable of receiving the participants, probably avoided transphobic practices.

We performed a qualitative analysis in order to evaluate the effect of stigma on the retention of TrTGW. This process is expressed, mainly, when social expectations based on heteronormativity conflict with gender identity and expression, leading to situations of discrimination and violence^47^. Moreover, other factors, such as social class and race/skin color, act together and exacerbate each other, reinforcing social inequalities and health disparities, with consequences even for access to health services^48^.

Although the qualitative approach brings challenges for the operationalization of phenomena such as gender, race/skin color, and class, which are incorporated in the analysis, it allows a more comprehensive understanding and better reading and interpretation of the research object, expanding the capacity to act and transform reality^49^.

The research team sought strategies to understand the reasons why some participants did not regularly engage in the intervention. In these cases, they sought ways to intervene, considering the particular characteristics and demands of the participants, even offering the possibility of changing their PN if necessary.

### Limitations

The choice of convenience sampling instead of methods traditionally used in studies with hard-to-reach populations, such as respondent-driven sampling (RDS) or snowball, in which pre-selected participants recruit others in order to constitute the desired sample, was a limitation of the study^50,51,52^. Since TransAmigas was a pilot project, aimed at assessing the feasibility and acceptability of peer navigation, and considering the limitation of resources and time, which resulted in a small sample, recruiting participants in the same health service would ensure the elimination of potential biases related to the organization and quality of care. Moreover, individuals with a recent HIV diagnosis who have not started or abandoned treatment are difficult to include in samples based on the participants’ own social relationship networks, such as RDS, or recruitment in spaces of sociability, such as time-location sampling (TLS). On the other hand, this option may have influenced the final sample, since a larger number of participants already linked to health services were recruited and they were possibly more likely to adhere to the treatment and procedures of the study.

## Conclusions

Identifying the factors associated with retention is essential to achieve more promising results. In the TransAmigas projects, besides the satisfactory results obtained, maintaining contact with the research team and having higher schooling level were associated with retention. Moreover, evidence shows that drug use may also play a role.

The strategies implemented, such as collective activities in public spaces, promoted experiences that increased the bonds among the team, the participants, and the PNs. Thus, these strategies should be included in the guidelines that contribute to strengthen retention.

Future studies with TrTGW should include contact at regular intervals, with additional efforts aimed at participants with lower schooling level. To measure and reduce potential losses, using monitoring tools that provide data in a timely manner is also important, helping adapt strategies of retention to the study. In line with the current literature, in the light of the gender affirmative model^30,31^, the multidimensionality of phenomena that can be harmful to health should be considered, seeking the support of existing intersectoral policies and encouraging the development of new policies, if necessary. Finally, showing the results of the studies to the populations involved in less formal environments, using accessible language, is important to engage and build community leaders and organizations that become part of the construction of solutions.

## Figures and Tables

**Table 1:** Sociodemographic, mental health, gender affirmation, and study design-related characteristics of *travestis* and transgender women with HIV. TransAmigas project, São Paulo, Brazil, 2018–2019 (n = 113).

Characteristics	n	%
**Sociodemographic**		
Age (years)		
18–24	23	20.4
25–34	50	44.3
≥ 35	40	35.4
Schooling level (completed years of study)		
< 12	73	64.6
≥ 12	40	35.4
Race/Skin color		
White	36	31.9
Black	13	11.5
Mixed-race	54	47.8
Other	10	8.8
Monthly per capita income (minimum wages) *		
< 1	42	37.2
1–2	31	27.4
> 2	40	35.4
Marital status		
Single or living alone	71	62.8
In a relationship	27	23.9
Married or living together	15	13.3
Housing situation		
Own house or apartment	19	16.8
Rented house or apartment	65	57.5
Living at work	8	7.1
Living in a shelter or foster home	13	11.5
Homelessness	4	3.5
Other	4	3.5
**Health data/Frequency of alcohol and illicit substance use in the last six months**		
HIV serostatus disclosure		
No	18	15.9
Yes	95	84.1
Psychological distress scale (K10)		
Likely to be well	24	21.2
Likely to have a mild disorder	19	16.8
Likely to have a moderate disorder	31	27.4
Likely to have a severe disorder	39	34.5
Frequency of alcohol use		
Never	31	27.4
Once a month or less	31	27.4
2–4 times a week	24	21.2
2–3 times a week	18	15.9
≥ 4 times a week	6	5.3
Daily	3	2.7
Illicit substance use in the last six months		
No	38	33.6
Yes	75	66.4
Suicide attempt		
No	68	60.2
Yes	45	39.8
**Experience of violence**		
Experience of verbal violence for being trans		
No	9	8.0
Yes	104	92.0
Experience of sexual violence for being trans		
No	62	54.9
Yes	51	45.1
**Gender affirmation**		
“Did you manage to change your name on your documents?”		
Yes, I did	29	25.7
I tried, but I couldn’t	2	1.8
I’m trying/I’ve already filed the lawsuit	12	10.6
I haven’t tried, but I intend to try	56	49.6
I haven’t tried	14	12.4
“My appearance matches my gender identity”?		
Strongly disagrees	1	0.9
Disagrees	4	3.5
Neither agrees nor disagrees	8	7.1
Agrees	71	62.8
Strongly agrees	29	25.7
**Study variables**		
Study type		
Control	38	33.6
Peer navigation intervention	75	66.4
Telephone contact at three months		
No	26	23.0
Yes	87	77.0

* Minimum wage value at the time of the research: BRL 954.00 (2018) and BRL 998.00 (2019).

**Table 2:** Bivariate analysis related to retention at nine months among *travestis* and transgender women with HIV. TransAmigas project, São Paulo, Brazil, 2018–2019 (n = 113).

Variables		Returned			OR	p-value	95%CI
	n (n)	%	yes (n)	%			
Age (years)						0.087	
< 35	26	35.6	47	64.4	1.00		
≥ 35	8	20.0	32	80.0	2.21		0.889–5.502
Schooling level (completed years of study)						0.012	-
< 12	28	38.4	45	61.6	1.00		-
≥ 12	6	15.0	34	85.0	3.53		1.313–9.468
Race/Skin color						0.615	-
White	8	22.2	28	77.8	1.00		-
Black	5	38.5	8	61.5	0.46	0.261	0.116–1.791
Mixed-race	18	33.3	36	66.7	0.57	0.257	0.216–1.504
Other	3	30.0	7	70.0	0.67	0.611	0.139–3.185
Monthly per capita income (in minimum wages)						0.563	-
< 1	14	33.3	28	66.7	1.00		-
≥ 1	20	28.2	51	71.8	1.28		0.559–2.906
In a relationship						0.488	-
No	23	32.4	48	67.6	1.00		-
Yes	11	26.2	31	73.8	1.35		0.577–3.155
Housing situation						0.128	-
Unstable	12	41.4	17	58.6	1.00		-
Stable	22	26.2	62	73.8	1.99		0.821–4.818
Probability of having a mental health disorder (K10 scale)						0.909	-
Likely to have a mild or moderate disorder	22	29.7	52	70.3	1.00		-
Likely to have a severe disorder	12	30.8	27	69.2	0.95		0.409–2.211
Frequency of alcohol use						0.496	-
Once a month or less	17	27.4	45	72.6	1.00		-
Twice a month or more	17	33.3	34	66.7	0.76		0.337–1.692
Illicit substance use in the last six months						0.059	-
No	7	18.4	31	81.6	1.00		-
Yes	27	36.0	48	64.0	0.40		0.155–1.033
Suicidal ideation or suicide attempt						0.847	-
No	20	29.4	48	70.6	1.00		-
Yes	14	31.1	31	68.9	0.92		1.424–4.043
Experience of verbal violence for being trans						0.825	-
No	3	33.3	6	66.7	1.00		-
Yes	31	29.8	73	70.2	1.18		0.276–5.01
Experience of sexual violence for being trans						0.275	-
No	16	25.8	46	74.2	1.00		-
Yes	18	35.3	33	64.7	0.64		0.284–1.431
Name change on personal documents						0.898	-
No	25	29.8	59	70.2	1.00		-
Yes	9	31.0	20	69.0	0.94		0.377–2.352
Gender affirmation score [mean = 0.0098; SD = 0.9078]					1.57	0.061	0.980–2.530
HIV serostatus disclosure						0.816	-
No	5	27.8	13	72.2	1.00		-
Yes	29	30.5	66	69.5	0.88		0.2856–2.683
Study type						0.497	-
Control	13	34.2	25	65.8	1.00		-
Peer navigation intervention	21	28.0	54	72.0	1.34		0.578–3.093
Telephone contact at three months						< 0.001	-
No	17	65.4	9	34.6	1.00		-
Yes	17	19.5	70	80.5	7.78		2.959–20.44

95%CI: 95% confidence interval; OR: odds ratio; SD: standard deviation.

**Table 3:** Multivariate model of retention at nine months among *travestis* and transgender women with HIV. TransAmigas project, São Paulo, Brazil, 2018–2019 (n = 113).

Variables	Retention at nine months					
	No		Yes		OR	95%CI
	n	%	n	%		
Age (years)						
< 35	26	35.6	47	64.4	1.00	-
≥ 35	8	20.0	32	80.0	1.42	0.50–4.04
Race/Skin color						
White	8	22.2	28	77.8	1.00	-
Black	5	38.5	8	61.5	0.90	0.17–4.73
Mixed-race	18	33.3	36	66.7	1.02	0.32–3.31
Other	3	30.0	7	70.0	0.90	0.16–5.08
Schooling level (completed years of study)						
< 12	28	38.4	45	61.6	1.00	-
≥ 12	6	15.0	34	85.0	3.26	1.02–10.42
Telephone contact at three months						
No	17	65.4	9	34.6	1.00	-
Yes	17	19.5	70	80.5	6.15	2.16–17.51
HIV serostatus disclosure						
No	5	27.8	13	72.2	1.00	-
Yes	29	30.5	66	69.5	0.87	0.22–3.49
Gender affirmation score					1.37	0.80–2.34

95%CI: 95% confidence interval; OR: odds ratio.

Note: pseudo R2 = 0.1880.
